# Editorial: Reviews in: pulmonary medicine 2023

**DOI:** 10.3389/fmed.2025.1589992

**Published:** 2025-03-27

**Authors:** Roberto G. Carbone, Francesco Puppo

**Affiliations:** Department Internal Medicine, University of Genoa, Genova, Italy

**Keywords:** pulmonary disease, interstitial lung disease, idiopathic pulmonary fibrosis, chronic obstructive pulmonary disease, lung cancer, pulmonary hypertension

Pulmonary disease is an ever-growing field. Recent research focuses on basic and clinical advances in interstitial lung disease (ILD) such as idiopathic pulmonary fibrosis (IPF) and the IPF genome, chronic obstructive pulmonary disease (COPD), epigenetics, pulmonary hypertension (PH), sarcoidosis, and eosinophilic pneumonia.

Autophagy plays a vital role in the maintenance of homeostasis and recent studies have explored the connection between autophagy and the development of different lung diseases. Autophagy appears to play a protective role in the development of IPF and a pathogenetic role in the progression of COPD. There are conflicting results on the role of autophagy in PH and asthma (1).

Epigenetic mechanisms include post-translational methylation and acetylation of histone proteins and DNA, and modulation of miRNA production. Up- or down-regulation of methylation in various genes have been demonstrated in COPD, and a variety of epigenetic alterations that include histone modification, DNA methylation, and non-coding RNA have been reported in IPF, and DNA methylation, histone modifications, and noncoding RNA have been shown in PAH (2). Additionally, epigenetics may improve early diagnosis and treatment of ILDs avoiding several biases, especially in the treatment of ILDs and in IPF the most common ILD with a poor survival rate.

A major current problem is the search for accurate vasodilator treatment in patients with pulmonary hypertension (PH) associated with IPF. The latter when combined with PH is regrouped in Group 3 of the ERS/ESC Guidelines (3). Nathan et al. (4) suggested treprostinil as an accurate vasodilator therapy in IPF. However, further clinical trials are needed to establish the efficacy of this treatment because the results of this study appear to be still inconclusive (5).

Multiplex immunolabeling and *in situ* sequencing transcriptomic analysis of lung tissue sections provided new insights into the immunopathogenesis of granulomas in sarcoidosis. Sarcoidosis lesions contain T-cell infiltrates that surround the granuloma core and B-cell clusters that are morphologically and molecularly suggestive of tertiary lymphoid structures (6). New perspectives on sarcoidosis have identified fatigue as an extremely significant symptom of the disease distinguishing it from other chronic diseases (7).

In terms of imaging [18F]-fluorodeoxyglucose positron emission tomography ([18F] FDG PET) is performed in suspected pulmonary and cardiac sarcoidosis. Recent data suggest that the association of [18F] FDG PET with late gadolinium tracer enhancement and cardiac magnetic resonance may help to differentiate cardiac sarcoidosis from other causes of myocardial inflammation (8).

Acute and chronic eosinophilic diseases are rare interstitial lung conditions. Pathological findings and high-resolution computed tomography (HRCT) imaging patterns may improve diagnostic accuracy and facilitate differential diagnosis with other eosinophilic lung diseases (9).

An international group of radiologists and pulmonologists (10) proposed a deep learning algorithm, based on the ATS/ERS/JRS/ALAT IPF guidelines criteria (SOFIA), to classify HRCT patterns for IPF diagnosis. The study included 203 patients with suspected IPF and concluded that artificial intelligence (AI) improves the accuracy of imaging evaluation and clinical diagnosis of ILD. Algorithms including clinical features, radiological patterns, and specific autoantibodies have been developed to improve the accuracy of diagnosis and management of ILD patients suffering from connective tissue diseases such as systemic sclerosis, rheumatoid arthritis, and polymyositis/dermatomyositis (11).

Patients affected by systemic sclerosis frequently develop interstitial lung disease (SSc-ILD). Patients with SSc-ILD have a significantly higher survival rate than patients with IPF-usual interstitial pneumonia (UIP). The survival rate of patients with SSc-ILD is negatively associated with the New York Heart Association (NYHA) class and pulmonary arterial pressure value (12).

There are still some limitations to the incidence and prevalence of ILDs reported in regional Registries. In fact, data from the Asian Pacific Registry have only recently been published allowing comparison with data from European Registries (13). ILD and IPF progression and survival remain uncertain and are currently associated with the number of exacerbations per year.

An association has been found between ILD and lung cancer in the same patients. These patients are affected by IPF and non-small cell lung cancer (NSCL); their prevalence is approximately 10% and they have a very poor prognosis. Of note, Uhlenbruch et al. (14) reported a 48% association between ILD and NSCLC at autopsy.

Last but not least, great progress has been made in IPF genomic analysis with the detection of well-established common and rare variants within the TERT and RTEL1 genes that, in combination with environmental factors, contribute significantly to IPF risk (15).

In conclusion, this annual review summarizes interesting advances in all forms of ILD and COPD; however, further clinical research in large multicenter clinical trials is needed in the near future to improve our understanding of these topics.
